# Stress Management in Elementary School Students: a Pilot Randomised Controlled Trial

**DOI:** 10.14806/ej.26.1.976

**Published:** 2021-08-23

**Authors:** Katerina Sofianopoulou, Flora Bacopoulou, Dimitrios Vlachakis, Ioulia Kokka, Evaggelos Alexopoulos, Liza Varvogli, George P. Chrousos, Christina Darviri

**Affiliations:** 1 Postgraduate Course of Science of Stress and Health Promotion, School of Medicine, National and Kapodistrian University of Athens, Athens, Greece; 2 University Research Institute of Maternal and Child Health & Precision Medicine and UNESCO Chair on Adolescent Health Care, National and Kapodistrian University of Athens, Aghia Sophia Children’s Hospital, Athens, Greece; 3 Laboratory of Genetics, Department of Biotechnology, School of Applied Biology and Biotechnology, Agricultural University of Athens, Athens, Greece; 4 Lab of Molecular Endocrinology, Center of Clinical, Experimental Surgery and Translational Research, Biomedical Research Foundation of the Academy of Athens, Athens, Greece

## Abstract

Research has shown that stress experiences begin in early stages of life. Stress management techniques have appeared to be beneficial for the development or enhancement of stress coping skills. The aim of this pilot randomised controlled trial was to assess the effect of a 12-week intervention, comprising training in diaphragmatic breathing and progressive muscular relaxation, on elementary school students’ stress levels. Outcomes on the quality of life and behavioural aspects of the students were also assessed. Standardised questionnaires were administered at baseline and after the 12-week intervention program. Fifty-two children aged 10 to 11 years were randomly assigned to intervention (n=24) and control groups (n=28). Children of the intervention group demonstrated lower levels of stress (in all three subscales of lack of well-being, distress, and lack of social support) and improved aspects of quality of life (physical, emotional, and school functioning). No significant differences were observed regarding the examined behavioural dimensions, in the intervention group. Larger randomised controlled trials with follow-up evaluations are needed to ascertain the positive outcomes of such programs on elementary school children.

## Introduction

Health-related habits start to form in early life stages, even before children can realise the impact of their choices on their health and quality of life (Petosa & Oldfield, 1985). Research shows that school children experience stress frequently and the main source are daily hassles (Bridley and Jordan, 2014). Researchers have noted that multiple daily hassles interact with one another and can have cumulative effects (Stansbury and Harris, 2000). These daily stressors emerge into a non-specific risk factor for a wide range of psychosomatic and behavioural problems, such as headaches, stomach aches, sleep difficulties (Berntsson et al., 2001), anxiety, depression, aggression, substance abuse, allergic or asthmatic attacks, withdrawal or outbursts, antisocial or disruptive behaviours (Van Praag and De Kloet, 2005).

Elevated stress levels can affect children’s mental and physical health (Garmezy and Rutter 1983). Child development and function *i.e.* academic performance (Blashill, 2016)can be affected by stressful family issues such as divorce (Amato, 2000).These observations reinforce the notion that educational efforts regarding coping strategies to relieve stress should begin in elementary school. Early stress coping education can help children experience less stress (Petosa and Oldfield, 1985) and maintain their coping skills in adulthood.

Stress management programs have positive health outcomes, such as reduced waking and evening cortisol, fasting blood sugar and resting heart rate (Pascoe et al., 2017) and beneficially effects on depression (Abbasian et al., 2014), social support (Baqutayan, 2011) and academic performance (Rentala et al, 2019). Several stress management programs have been implemented in healthy and non-healthy children populations; individual psychotherapy (March, 1998), family therapy (Bernardon, 2010), specific cognitive and behavioural strategies such as empowerment techniques (Carlier, 2020), all with positive effects on stress levels. Two of the techniques commonly taught in stress management educational programs are diaphragmatic breathing and progressive muscular relaxation (PMR). Diaphragmatic breathing is an efficient body-mind stress reduction method. It is considered to help in emotional regulation and social adaptation (Porges, 2001). Regarding PMR, it is a deep relaxation technique which is based on the simple practice of tensing one muscle group at a time followed by a relaxation phase with release of the tension (Jacobson, 1987). So far, health psychology and clinical studies have used these techniques in a complementary way (Chen et al., 2017; Tsang et al., 2006) and not independently from other stress management techniques.

The aim of this study was to investigate the effect of a twelve-week intervention program of diaphragmatic breathing and progressive muscle relaxation on the subjective stress levels of elementary school children. Secondary aims were to investigate the effect of the program on the children’s quality of life and behavioural aspects.

## Materials, Methodologies and Techniques

### Study design and participant recruitment

This randomised, two-armed, pilot study was conducted in children of the 5th grade of a private elementary school in Athens, Greece over a three-month period. Eligible students should be able to write and read in Greek, not have any chronic medical condition or receive systematic medication or any type of psychological support. Study participants were randomised into two groups; the intervention group which received diaphragmatic breathing and progressive muscular relaxation training and the control group. Assignment of children to either the intervention group or the control group was based on random numbers generated by an online random number generator^1^.

### Ethical considerations

The study protocol was consistent with the Declaration of Helsinki and was approved by the ethics committee of the Medical School of the National and Kapodistrian University of Athens in Greece. All children and their parents or guardians were informed about the study’s procedure and goals. Parental/guardian signed consent was mandatory for a child to participate in the study. All participants had the right to interrupt their participation at any step of the study, without any consequences for them.

### Measurements

#### Stress in Children Questionnaire (SiC):

This is a 21-item self-report questionnaire measuring subjective stress levels in childhood. It measures three subscales: lack of well-being, distress and lack of social support. The instrument demonstrates good psychometric properties and high internal consistence, with a Cronbach’s α coefficient of 0.86 (Osika et al., 2007).

#### Strengths and Difficulties Questionnaire (SDQ):

This is a 25-item behavioural instrument, measuring 5 different dimensions; emotional symptoms, conduct problems, hyperactivity/inattention, peer relationship problems and pro-social behaviour. In community samples, the SDQ can predict the presence of a psychiatric disorder with good specificity and moderate sensitivity (Goodman, 1997;Giannalopoulos et al., 2009)

#### Pediatric Quality of Life (PedsQL) Inventory:

The 23-item Peds QL was designed to measure the core dimensions of health as delineated by the World Health Organization (physical, emotional, social, and school functioning). It measures health-related quality of life in children and adolescents either healthy or with acute and chronic health conditions (Papaevangelou et al., 2007; Varni et al., 1999).

#### Intervention:

A total of 12 weekly sessions were delivered by KS (MSc in stress management) and CD (Professor of stress management and health promotion) to the intervention group. During the first session, participants of both groups completed the questionnaires and received the same information about stress and its effects on health. They were also informed about the importance of a healthy lifestyle (healthy diet, regular physical activity and sleeping routine). In the following 10 sessions participants of the intervention group were trained on and practiced diaphragmatic breathing and progressive muscular relaxation and received stres-related psycho-education. At the same time the control group, located in a different classroom, had leisure activities under their teachers’ supervision. During the last session, final assessments were made for both groups by re-administering the questionnaires.

### Statistical analysis

The baseline group characteristics are presented as means, standard deviations (SD), absolute and proportion values. Pearson’s chi square and Fisher’s exact tests were performed for frequency group comparisons; the independent two-sample Student’s t-test for means differences between the groups and the paired Student’s t-test for means differences within the groups. Their equivalent non-parametric tests (Mann–Whitney U test and Wilcoxon signed-rank test) were implemented in case the hypothesis of normality was violated. The normality of data was assessed with the Shapiro-Wilk test and the normal probability plots (Q-Q plots, P-P plots). All statistical tests applied were two-tailed using the <0.05 level of statistical significance. All statistical analyses were conducted using the statistical software package StataSE (V. 10, Data Analysis and Statistical Software, Stata Corp LP, Texas, USA; 2009).

## Results

**Figure 1.**
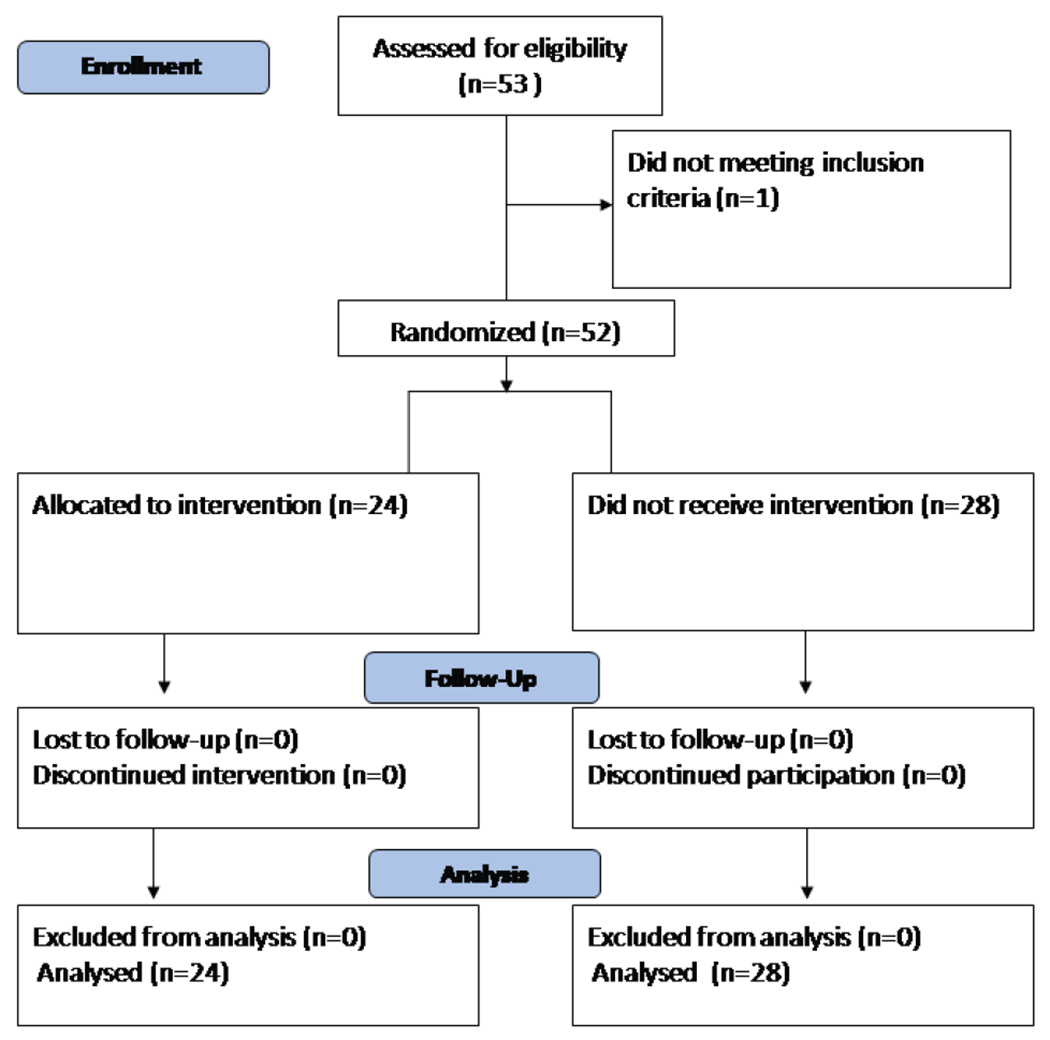
Participants’ flow diagram.

A total of 52 students (31 females and 21 males) participated in the study. The flow diagram of the study is illustrated in Figure 1.

No dropouts occurred in either group. Given that participants of both groups were of the same school grade, their age was between 10 and 11 years. No significant difference was found between groups regarding the sex. The age and sex characteristics of each group are outlined in Table 1.

Baseline psychometric characteristics of the study groups are presented in Table 2.

As shown in Table 3, when the psychometric characteristics of the two groups after the intervention were compared, statistically significant differences were found in all three subscales of the SiC questionnaire in favor of the intervention group. There were no significant differences between the two groups after the intervention in the other quality of life and behavioural parameters.

Within group comparisons pre- and post-intervention are shown in Tables 4 and 5 for the intervention and control groups, respectively. Statistically significant increases of the PedsQL physical, emotional, and school functioning scores were found for the intervention group. Statistically significant lower scores were found for the same group in all subscales of the SiC questionnaire. Interestingly, for the control group, the score of the distress subscale of the SiC, the SDQ total score and conduct problems subscale score were significantly reduced, while the PedsQL social functioning score was significantly increased, at the end of the study.

## Discussion

This pilot study examined the effects of a stress management interventional program, using the techniques of diaphragmatic breathing and PMR, to reduce stress in elementary students. Results showed a significant impact of the program on the subjective stress levels, with improvements in all three subscales *i.e.*, lack of social support, distress, and lack of well-being.

Results of this study showed that diaphragmatic breathing and PMR can result in reduction of the children’s distress levels. This finding is in line with the study of Ma *et al.*, who measured saliva levels of the stress hormone cortisol, after the implementation of an 8-week diaphragmatic breathing program on healthy individuals(Ma et al., 2017). Even when referring to non-healthy population, these techniques have been proven beneficial regarding stress reduction. A systematic review of the influence of PMR on patients with schizophrenia showed that the technique can reduce psychological distress and anxiety (Vancampfort et al., 2013). Wilk and Turkoski (2001) reported the same positive results on cardiac patients. The researchers implemented a stress management program based on PMR in a cardiac rehabilitation facility and outcomes included lower heart rate, improved sleep quality and lower levels of anxiety, all characteristics of low stress levels (Wilk and Turkoski, 2001). Although the aforementioned studies concerned adults, positive outcomes have been reported in studies of minor subjects as well. A meta-analysis of school stress management programs that included diaphragmatic breathing and PMR, demonstrated significantly positive results in stress reduction (Kraag et al., 2006).

This study examined the effect of the implemented program on the students’ perception of social support and well-being, which are stress-related dimensions. Results showed that students improved their perception of social support and their sense of well-being. High social support seems to facilitate stress coping and enhances well-being (Charney and Southwick, 2007). Numerous studies have shown that long term stress and lack of social support and well-being go hand in hand with poor mental health(Mohr and Classen 2004; Michalak et al., 1999; Paykel, 1994; Toussaint et al., 2016).

This study confirmed the beneficial effects of diaphragmatic breathing and PMR on students’ emotional functioning. According to the American Psychological Association the average stress level of the U.S. population in 2015 was 5.1 in a scale of 1 to 10 (APA, 2015). Stress is related to discomforting emotional symptoms such as feelings of depression, anxiety, irritability, and low self-efficacy. The significant improvement in emotional functioning in this pediatric study is in line with previous research concerning the effects of stress management programs on the psychological symptoms of stress (Chiesa and Serretti, 2009).

The implemented program resulted in a significant improvement in the school functioning level of the students. These results contradict recent findings on the relation of cortisol to school functioning of children, where no correlation was demonstrated between cortisol levels and school behaviour (Simons et al., 2017). This disagreement could be explained by the ongoing development of the cortisol circadian rhythm in childhood.

This study has some limitations. The main limitation is the lack of biological measurements for stress *i.e.*, in blood, urine or saliva (Dhama et al., 2019) and the use of solely self-report questionnaires. Nevertheless, this was a low-cost intervention, easy to implement in a school setting. The lack of long-term follow-up, the small sample size and the limited age range of the participants do not allow generalization of the results. More complex, longitudinal interventions are required for children to adopt new, healthier behavioural patterns with strong stress coping skills (Baranowski et al., 2003) and to attain concrete results regarding the effect of diaphragmatic breathing and PMR on children’s stress levels.

## Figures and Tables

**Table 1.** Study groups’ characteristics.

	Intervention group	Control group	p-value
**Sex**	N=24 (%)	N=28 (%)	0.337
**Female**	16 (66.67)	15 (53.57)	
**Male**	8 (33.33)	13 (46.43)	

**Table 2.** Baseline psychometric characteristics of study groups.

	Intervention Group Mean ± SD	Control group Mean ± SD	p-value*

**SDQ score**			
Total	18.11 ± 7.37	18 ± 6.47	0.952
Emotional symptoms	3.26 ± 2.60	2.92 ± 2.72	0.574
Conduct problems	1.48 ± 1.80	1.40 ± 1.83	0.852
Hyperactivity score	4.25 ± 2.90	3.82 ± 3.37	0.460
Peer problem	3.30 ± 2.81	2.5 ± 2.08	0.361
Prosocial behaviour	6.37 ±2.13	7.35 ± 2.45	0.117

**PedsQL score**			
Physical functioning	66.20 ± 28.10	70.75 ± 23.07	0.560
Emotional functioning	56.30 ± 30.21	56.96 ± 26.46	0.773
Social functioning	67.03 ± 33.63	67.50 ± 28.90	0.530
School functioning	66.30 ± 34.57	69.46 ± 26.60	0.852

**SiC score**			
Total	46.57 ± 5.86	43.85 ± 8.82	0.247
Lack of well-being	15.17 ± 4.00	14.08 ± 3.91	0.344
Distress	17.70 ± 3.36	18.20 ± 2.87	0.584
Lack of social support	14.34 ± 2.44	13.11 ± 2.98	0.123

SDQ: Strengths and Difficulties Questionnaire, PedsQL: Pediatric Quality of Life, SiC: Stress in Children

* Difference of frequencies tested with Pearson’s chi square or Fisher’s exact test, difference of means with Student’s t-test and non-parametric Mann-Whitney U test (applied in mean weight, mean SDQ emotional symptoms, conduct problems, hyperactivity, peer problem scores, in all PedsQL scores and SiC distress sub-score).

**Table 3.** Comparison of the psychometric characteristics of the two groups after the intervention.

	Intervention Group Mean ± SD	Control group Mean ± SD	p-value*

**SDQ score**			
Total	15.74 ± 6.00	14.35 ± 6.34	0.410
Emotional symptoms	2.51 ± 2.27	1.92 ± 1.70	0.387
Conduct problems	1.30 ± 1.61	0.82 ± 1.21	0.317
Hyperactivity score	3.30 ± 2.78	3.42 ± 3.24	0.878
Peer problem	2.96 ± 2.54	1.96 ± 1.55	0.225
Prosocial behaviour	6.14 ± 2.20	7.17 ± 2.31	0.096

**PedsQL score**			
Physical functioning	75.81 ± 25.40	76.00 ± 18.90	0.654
Emotional functioning	68.88 ± 25.00	65.00 ± 24.50	0.314
Social functioning	73.15 ± 28.76	77.85 ± 16.75	0.986
School functioning	77.22 ± 25.12	74.46 ± 23.50	0.401

**SiC score**			
Total	37.64 ± 5.29	42.26 ± 8.06	0.019
Lack of well-being	11.28 ± 3.57	13.53 ± 4.05	0.037
Distress	14.52 ± 2.51	16.11 ± 2.42	0.050
Lack of social support	11.24 ± 2.31	12.75 ± 2.68	0.034

SDQ: Strengths and Difficulties Questionnaire, PedsQL: Pediatric Quality of Life, SiC: Stress in Children

* Difference of means tested with Student’s t-test and non-parametric Mann-Whitney U test (applied in mean SDQ emotional symptoms, conduct problems, hyperactivity, peer problem scores, in all PedsQL scores and in SiC distress sub-score). Statistical significance at p<0.05.

**Table 4.** Comparisons of the psychometric characteristics of the intervention group pre- and post-intervention.

	Before Intervention Mean ± SD	After Intervention Mean ± SD	p-value*

**SDQ score**			
Total	18.11 ± 7.37	15.74 ± 6.00	0.131
Emotional symptoms	3.26 ± 2.60	2.51 ± 2.27	0.069
Conduct problems	1.48 ± 1.80	1.30 ± 1.61	0.666
Hyperactivity score	4.25 ± 2.90	3.30 ± 2.78	0.069
Peer problem	3.30 ± 2.81	2.96 ± 2.54	0.402
Prosocial behaviour	6.37 ± 2.13	6.14 ± 2.20	0.550

**PedsQL score**			
Physical functioning	66.20 ± 28.10	75.81 ± 25.40	0.001
Emotional functioning	56.30 ± 30.21	68.88 ± 25.00	0.003
Social functioning	67.03 ± 33.63	73.15 ± 28.76	0.226
School functioning	66.30 ± 34.57	77.22 ± 25.12	0.029

**SiC score**			
Total	46.57 ± 5.86	37.64 ± 5.29	< 0.001
Lack of well-being	15.17 ± 4.00	11.28 ± 3.57	0.001
Distress	17.70 ± 3.36	14.52 ± 2.51	< 0.001
Lack of social support	14.34 ± 2.44	11.24 ± 2.31	< 0.001

SDQ: Strengths and Difficulties Questionnaire, PedsQL: Pediatric Quality of Life, SiC: Stress in Children

* Difference of means tested with two dependent samples Student’s t test or nonparametric Wilcoxon signed rank test (implemented in SDQ emotional symptoms, conduct problems, hyperactivity, peer problem scores, in all PedsQL scores and in SiC score). Statistical significance at p<0.05.

**Table 5.** Comparison of the psychometric characteristics of the control group pre- and post- intervention.

	Before Intervention Mean ± SD	After Intervention Mean ± SD	p-value*

**SDQ score**			
Total	18 ± 6.47	14.35 ± 6.34	0.007
Emotional symptoms	2.92 ± 2.72	1.92 ± 1.70	0.062
Conduct problems	1.40 ± 1.83	0.82 ± 1.21	0.013
Hyperactivity score	3.82 ± 3.37	3.42 ± 3.24	0.498
Peer problem	2.5 ± 2.08	1.96 ± 1.55	0.114
Prosocial behaviour	7.35 ± 2.45	7.17 ± 2.31	0.672

**PedsQL score**			
Physical functioning	70.75 ± 23.07	76.00 ± 18.90	0.212
Emotional functioning	56.96 ± 26.46	65.00 ± 24.50	0.121
Social functioning	67.50 ± 28.90	77.85 ± 16.75	0.049
School functioning	69.46 ± 26.60	74.46 ± 23.50	0.166

**SiC score**			
Total	43.85 ± 8.82	42.26 ± 8.06	0.430
Lack of well-being	14.08 ± 3.91	13.53 ± 4.05	0.830
Distress	18.20 ± 2.87	16.11 ± 2.42	0.002
Lack of social support	13.11 ± 2.98	12.75 ± 2.68	0.500

SDQ: Strengths and Difficulties Questionnaire, PedsQL: Pediatric Quality of Life, SiC: Stress in Children

* Difference of means tested with two dependent samples Student’s t test or nonparametric Wilcoxon signed rank test (implemented in SDQ emotional symptoms, conduct problems, hyperactivity, peer problem scores, in all PedsQL scores and in SiC score). Statistical significance at p<0.05.

## Key Points

- Stress experiences begin in early stages of life.
- An elementary school program on stress coping skills was implemented for 12 weeks.
- Diaphragmatic breathing and progressive muscular relaxation reduced students’ subjective stress levels.
